# Differences in Phenological Estimation From Multi‐Vegetation Indices Across the Yellow River Basin

**DOI:** 10.1002/ece3.73601

**Published:** 2026-04-29

**Authors:** Qinyue Yu, Yan Bai, Juanle Wang

**Affiliations:** ^1^ Institute of Geographic Sciences and Natural Resources Research Chinese Academy of Sciences Beijing China; ^2^ University of Chinese Academy of Sciences Beijing China; ^3^ Jiangsu Center for Collaborative Innovation in Geographical Information Resource Development and Application Nanjing China

**Keywords:** autumn senescence, EVI, kNDVI, land surface phenology, PPI

## Abstract

Satellite‐derived vegetation indices (VIs) are widely used to monitor land surface phenology (LSP). Plant Phenology Index (PPI) and kernel NDVI (kNDVI) have emerged as promising tools for improving accuracy of LSP estimation. However, differential phenological performance of these two indices relative to traditional ones remains unclear. Here, we evaluated consistency of PPI, kNDVI, and EVI derived from MODIS against solar‐induced chlorophyll fluorescence (SIF) and flux tower GPP data, and investigated spatiotemporal changes in retrieving four phenological metrics across the Yellow River Basin (YRB). Results indicated that EVI exhibited a stronger overall correlation with SIF, whereas PPI achieved the best performance for GPP estimation (*R* ^2^ = 0.76, RMSE = 1.31 g C m^−2^ day^−1^), particularly in snow‐affected alpine regions. PPI also showed more balanced and stable late‐season errors. Pronounced temporal discrepancies were detected in downturn date (DD) and recession date (RD) among three VIs. PPI demonstrated closer alignment with SIF and GPP during the autumn decline and consistently identified both the onset and termination of autumn senescence approximately 25–50 days earlier than kNDVI and EVI. These variations were particularly evident in DD, where kNDVI and EVI indicated delays (0.247 days year^−1^ and 0.038 days year^−1^, respectively), while PPI showed an advance (−0.120 days year^−1^). In addition, widespread advances were observed in upturn date (UD) and stabilization date (SD), primarily in the transitional zones between forest and grassland sub‐regions, along with significant delays in the RD across the west‐central region of basin. Furthermore, 70% of the YRB exhibited an advancing trend in the DD of PPI, which contrasted distinctly with the trends observed for kNDVI and EVI. These findings suggest improved performance of PPI in detecting autumn senescence and provide valuable insights into the potential of various VIs for LSP retrievals.

## Graphical abstract

This study evaluated the differential performance of PPI, kNDVI, and EVI in monitoring land surface phenology across the Yellow River Basin. The three vegetation indices showed pronounced divergence in capturing autumn phenological metrics, particularly the downturn date (DD) and recession date (RD). Among them, PPI demonstrated greater sensitivity to autumn phenology compared with kNDVI and EVI.

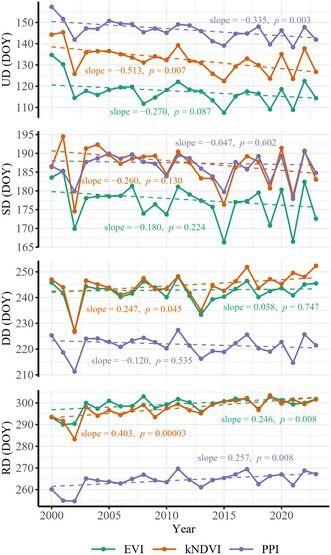

## 1 Introduction

Vegetation phenology, the cyclical recurrence of plant life events such as sprouting, leaf unfolding, flowering, and senescence (Lieth 1974), serves as a sensitive barometer of how terrestrial ecosystems respond to climate change (Piao et al. 2007). By quantifying the timing of these events, researchers can trace the biosphere's response to intra‐annual and inter‐annual fluctuations in climatic, hydrological, soil, and anthropogenic factors (White et al. 1997). As climate change intensifies, significant shifts in vegetation phenology have been observed over the past decades (Körner and Basler 2010). Accurate and continuous monitoring of phenological dynamics is therefore essential, not only for detecting the impacts of environmental changes on terrestrial ecosystems (Moon et al. 2019), but also for improving models related to vegetation variations, biogeochemical cycles, and climate forecasting (Yuan, Zhao, et al. 2020).

Satellite observations have rapidly evolved from a powerful supplement to ground‐based observations into an indispensable tool for monitoring land surface phenology (LSP) across regional to global scales (Smith et al. 2018), largely due to its extensive spatial coverage, high temporal sampling frequency, and the availability of over three decades worth of time series data (Liu, Wu, Liu, et al. 2017). A variety of satellite‐derived vegetation indices (VIs) have been adopted as LSP indicators for capturing seasonal dynamics of leaf and canopy structure. The Normalized Difference Vegetation Index (NDVI) is prevalently used in phenological investigations across diverse scales, primarily owing to its straightforward calculation and long‐term observational records exceeding 30 years (Camps‐Valls et al. 2021). However, NDVI is vulnerable to atmospheric conditions, soil background effects in areas with sparse vegetation cover (Balzarolo et al. 2016), and it is susceptible to saturation in multi‐layered and thick canopies (Huete et al. 2002). The Enhanced Vegetation Index (EVI) was developed to mitigate these drawbacks by injecting blue‐band information and a semi‐empirical canopy‐background correction (Huete et al. 2002). Nevertheless, EVI has been reported to be sensitive to snow, resulting in unreliable values at high northern latitudes during winter (Schubert et al. 2012). Recent years have witnessed the development of novel VIs. Jin and Eklundh (2014) designed the Plant Phenology Index (PPI) directly from rigorous radiative transfer equations. This index can effectively minimize the influence of soil and snow backgrounds, and it is almost linearly related to the canopy Leaf Area Index (LAI), which is viewed as a fundamental property of phenology‐specific VIs (Fang et al. 2019; Liu et al. 2024). Parallel advances in machine learning led Camps‐Valls et al. (2021) to propose the kernel‐based NDVI (kNDVI). The kNDVI shows superior resistance to saturation effects and intricate phenological cycles while exhibiting enhanced robustness to seasonal variations. Compared to NDVI and the near‐infrared reflectance of vegetation (NIRv), kNDVI showed stronger correlations with Gross Primary Productivity (GPP) and Solar‐Induced chlorophyll Fluorescence (SIF) (Camps‐Valls et al. 2021). GPP directly reflects the intensity of vegetation photosynthesis (Xia et al. 2015), and SIF is a direct by‐product of this process (Lu et al. 2018). Therefore, comparing the correlations among different VIs, GPP, and SIF provides a robust basis for evaluating the performance of VIs‐derived phenology extraction (Duveiller et al. 2020).

The emerging VIs provides a new perspective on phenology extraction and offers the potential to enhance LSP monitoring accuracy. Yet, exploration of their potential in LSP detection remains limited. Prior researches have documented that PPI can be robustly applied to large‐scale phenological estimation in the Northern Hemisphere (Karkauskaite et al. 2017), semi‐arid ecosystems of Africa (Abdi et al. 2019), and particularly high‐latitude regions of Northern Europe (Jin et al. 2017; Tian et al. 2021; Junttila et al. 2023). Meanwhile, studies using kNDVI for phenology extraction have typically focused either on a single crop (i.e., summer corn) (Han et al. 2025), or on an isolated vegetation type (i.e., grassland) (Bellini et al. 2023). Moreover, different VIs tend to highlight distinct phenological characteristics and exhibit varying regional applicabilities. Zhang et al. (2022) indicated that NIRv and SIF outperformed both NDVI and EVI in retrieving phenological information in eastern China. Similarly, Wang et al. (2022) showed that SIF and NIRv are able to capture complementary aspects of dryland vegetation productivity more effectively than NDVI or kNDVI. Conversely, kNDVI performs better than NIRv and NDVI in dense tropical canopies and arid regions (Camps‐Valls et al. 2021). PPI is found to be superior to NDVI and EVI for estimating the start of the growing season across boreal regions in the Northern Hemisphere (Karkauskaite et al. 2017). Comparison of multiple VIs is imperative for accurate LSP estimation and deeply understanding of complex relationships between ecosystem processes and climate change (Walker et al. 2014; Wu et al. 2014). However, a pivotal gap in investigating the adaptability of novel and traditional VIs‐based LSP estimation comparatively across diverse ecosystems at regional scales is exposed.

The Yellow River Basin (YRB) is an important ecological barrier in China, and is a key region implemented vegetation restoration projects for decades. The pronounced topographic gradients, diverse geomorphic settings, and heterogeneous hydrothermal conditions together support a broad spectrum of vegetation types across the region (Wohlfart et al. 2016). It is also recognized as a prominent vegetation greening area that has experienced unprecedented impacts of both climate change and human activities (Piao et al. 2019). Therefore, the YRB stands as an ideal region to analyze the interaction between ecosystem and environmental change. In this study, we calculate two novel VIs (i.e., PPI and kNDVI) and a traditional VI (i.e., EVI) from Moderate Resolution Imaging Spectroradiometer (MODIS), and aims to (1) examine the consistency of PPI, kNDVI, and EVI in tracking seasonal variations of GPP using SIF and ground‐based observations, (2) compare the performance of three different VIs in retrieving phenological metrics across the YRB and vegetation sub‐regions, and (3) explore the potential for these VIs to complement each other in extracting phenological information.

## 2 Materials and Methods

### 2.1 Study Area

**FIGURE 1:**
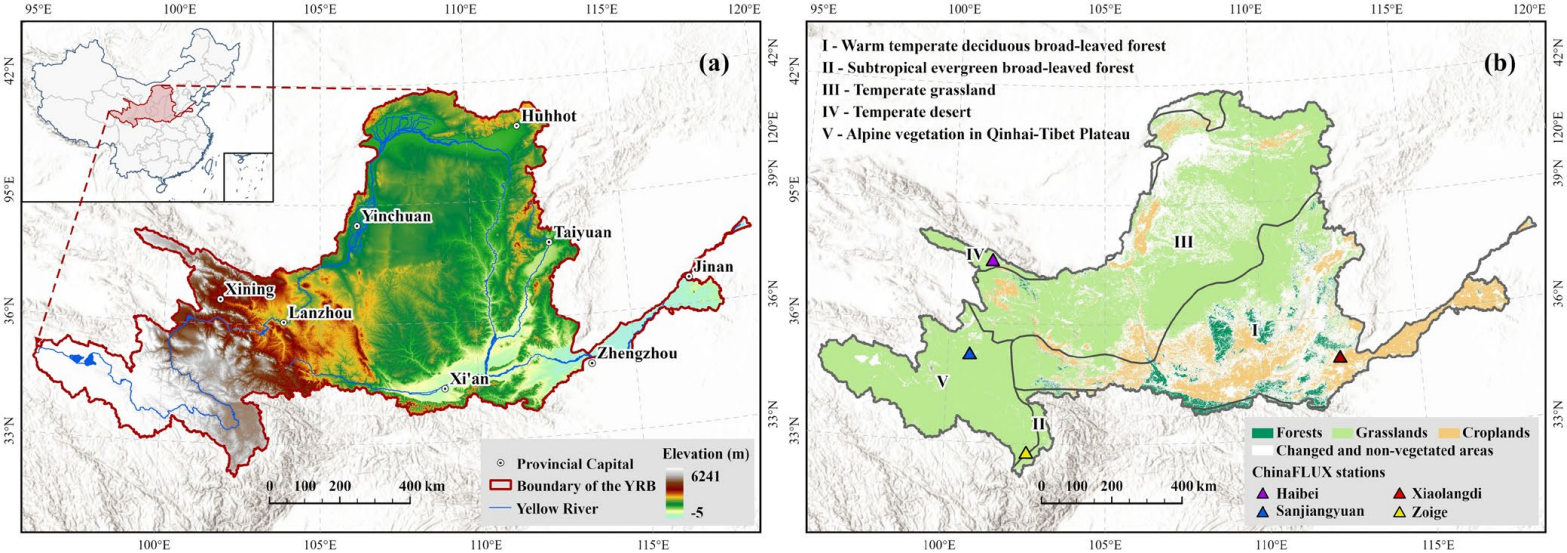
(a) Geographic location and elevation, (b) spatial distribution of carbon flux stations, vegetation sub‐regions, and vegetated land cover types of the YRB.

The YRB (95°53′‐119°05′ E, 32°10′‐41°50′ N), the second‐largest river basin in China, spans across three major plateaus (i.e., the Qinghai‐Tibet Plateau, the Inner Mongolia Plateau, and the Loess Plateau) and the North China Plain. It covers an expansive area of roughly 795,000 km^2^. The terrain in the YRB varies significantly, which is high in the west (above 4000 m) and low in the east (below 100 m) (Figure 1a). This basin is located in a transitional zone between the southeast monsoon climate and the northwest inland arid climate, resulting in distinct climatic differentiation characteristics. Over the past six decades, the average annual temperature is about 9.4°C with an increase from north to south, while the mean annual precipitation is approximately 466.6 mm, predominantly occurring in summer and decreasing from southeast to northwest (Wang et al. 2021). Owing to its diverse topography and uneven hydrothermal conditions, five vegetation sub‐regions have been identified in the YRB, including: (I) warm temperate deciduous broad‐leaved forest, (II) subtropical evergreen broad‐leaved forest, (III) temperate grassland, (IV) temperate desert, and (V) alpine vegetation in the Qinghai‐Tibet Plateau. Figure 1b shows that sub‐region I is dominated by forests and croplands, and the other four sub‐regions are primarily characterized by grasslands.

### 2.2 Data Sources

Three VIs, including PPI, kNDVI, and EVI, were computed from the MODIS Nadir Bidirectional Reflectance Distribution Function (BRDF)‐Adjusted Reflectance (NBAR) daily product (MCD43A4, v061, 500 m) available on Google Earth Engine (Schaaf and Wang 2021). To eliminate the effects of atmospheric interference and snow cover noises on MCD43A4 data, the corresponding MCD43A2 product flagging quality assurance information was used to filter the daily reflectance data at each pixel, and then pixels with good quality were averaged to an 8‐day temporal scale (Huang et al. 2024). In addition, data gaps covering the first 57 days of 2000 in the MCD43A4 were substituted with the average values from the identical period during 2001–2023.

A high spatial resolution SIF dataset in China (HCSIF) was acquired from Tao et al. (2024). This downscaled dataset was generated from the TROPOspheric Monitoring Instrument (TROPOMI) aboard Sentinel‐5P, integrated with other auxiliary data, such as spectral reflectance and land surface temperature of MODIS, as well as meteorological and topographic data, using a weighted stacking algorithm. The HCSIF dataset, spanning the periods from 2000 to 2020, provides a spatial resolution of 500 m and a temporal resolution of 8 days, which aligns with the spatiotemporal resolution of three VIs. The in situ GPP data of four flux towers used in this study were collected from the Chinese Terrestrial Ecosystem Flux Research Network (ChinaFLUX, https://www.chinaflux.org/), a long‐term network that relies on the Chinese Ecosystem Research Network (CERN) and provides long‐term eddy covariance observations of carbon, water, and energy exchanges across major terrestrial ecosystems in China (Yu et al. 2006). The selected sites include Xiaolangdi (deciduous broadleaved forest), Sanjiangyuan (grassland), Zoige (alpine meadow), and Haibei (alpine meadow). Brief information about each site is available in Table 1.

**TABLE 1:** Characteristics of four selected ChinaFlux sites in this study.

Site name	Latitude (°E)	Longitude (°N)	Altitude (m)	Vegetation type	Years
Xiaolangdi	35.02	112.47	410	Forest	2011–2020
Sanjiangyuan	34.35	100.48	3950	Grassland	2012–2016
Zoige	32.97	102.62	3465	Grassland	2015–2020 (missing data in 2019)
Haibei	37.62	101.30	3205	Grassland	2015–2020

The MODIS land cover products with a spatial resolution of 500 m (MCD12Q1, v061) were used to identify unchanged vegetation types in the YRB during 2001–2023 (Friedl and Sulla‐Menashe 2022). These data were downloaded from NASA Earth Observing System Data and Information System (EOSDIS, https://www.earthdata.nasa.gov/), based on the International Geosphere‐Biosphere Program (IGBP) classification scheme. To ensure consistency in phenological estimation, this study confined comparative analysis to the 23 years of stable vegetated areas, which were reclassified into forest (including shrubland), grassland, and cropland as shown in Figure 1b. Vegetation sub‐region data in shapefile format was obtained from the National Earth System Science Data Center of China (NESSDC, http://www.geodata.cn/).

### 2.3 LSP Metrics Extraction

#### 2.3.1 Vegetation Indices Calculation

The EVI is formulated to optimize the vegetation signal in high biomass regions, and to enhance the capability of atmospheric resistance by integrating the blue band to correct for aerosol effects in the red bed (Huete et al. 2002). It is computed as follows:

(1) $$\mathrm{EVI}=G\times \frac{\rho_{\mathrm{NIR}}-\rho_{\mathrm{red}}}{\rho_{\mathrm{NIR}}+C_{1}\times \rho_{\mathrm{red}}-C_{2}\times \rho_{\text{blue}}+L}$$

where $\rho_{\mathrm{NIR}}$, $\rho_{\mathrm{red}}$, and $\rho_{\text{blue}}$ are the MCD43A4 surface reflectance in near‐infrared (NIR, 841–876 nm), red (620–670 nm), and blue (459–479 nm) bands, respectively; *G* is the gain factor, *L* is the canopy background adjustment factor, and *C*_1_ and *C*_2_ are aerosol resistance coefficients. The parameters *G*, *L*, *C*_1_, and *C*_2_ were respectively set to 2.5, 1, 6, and 7.5 according to Huete et al. (2002).

The kNDVI is a robust proxy for ecosystem productivity, exhibiting greater resistance to saturation, bias, and complex phenological cycles. It is calculated by exploiting a kernel function that considers all higher‐order relationships between the NIR and red (RED) bands, rather than merely the first‐order linear ones (Camps‐Valls et al. 2021):

(2) $$\text{kNDVI}=\tanh\left({\left(\frac{\mathrm{NIR}-\mathrm{RED}}{2\sigma }\right)}^{2}\right)$$

where *σ* is a length‐scale parameter specified for each particular application. It quantifies the index's sensitivity to vegetation density. An optimal choice is to use the average value $\;\sigma =0.5\left(\mathrm{NIR}+\mathrm{RED}\right)$ as recommended by Camps‐Valls et al. (2021).

The PPI is a physically based spectral index designed to retrieve plant phenology. It is designed to be linearly related to LAI, given a certain soil background. PPI is derived from the solution to a radiative transfer equation based on modified Beer's Law for canopy reflectance, using NIR and red reflectance bands (Jin and Eklundh 2014). PPI (unit: m^2^ m^—2^) is calculated according to the following equations:

(3) $$\mathrm{PPI}=-K\times \ln\left(\frac{M-\mathrm{DVI}}{M-\mathrm{DV}I_{s}}\right)$$

where DVI (Difference Vegetation Index) is the difference between NIR and red reflectance from MCD43A4, M is a site‐specific canopy maximum DVI, $\mathrm{DV}I_{s}$ is the DVI of bare soil, and *K* is a gain factor dependent on solar zenith angle θ, representing a geometric function of leaf angular distribution and the instantaneous diffuse fraction of solar radiation. K is calculated as (Jin and Eklundh 2014):

(4) $$K=\frac{0.25\cos\left(\theta \right)}{\left(1-d_{c}\right)G+d_{c}\cos\left(\theta \right)}\cdot \frac{1+M}{1-M}$$

where θ is a solar zenith angle for clear‐sky and standard atmospheric conditions, $d_{c}$ is an instantaneous diffuse fraction of solar radiation at solar zenith angle θ, and G is a geometric function of leaf angle distribution. According to Wingate et al. (2015), $\mathrm{DV}I_{s}$ = 0.09 and *G* = 0.5 were used, and M was computed as the maximum DVI value in the 24‐year time series for each pixel in this study.

#### 2.3.2 Phenological Metrics Retrieval

The R package *phenofit* proposed by Kong et al. (2022) was applied to extract LSP metrics from PPI, kNDVI, and EVI. To ensure comparability among the phenological results, an initial rough fitting was performed on three VIs time series to capture the seasonal signal and facilitate the division of growing seasons. The weighted HANTS (*wHANTS*) function was chosen for this rough fitting due to its distinctive harmonic analysis technique, which effectively preserves the periodic structure of data during smoothing (Yang et al. 2015; Bush et al. 2017). Subsequently, fine curve fitting was implemented to reconstruct daily VIs time series within the growing season, enabling the detection of rapid vegetation changes throughout each season. The Beck function was selected as the preferred fine curve fitting method in this study, as it yields smoother and more stable fitting results with superior computing efficiency compared to piecewise models (Beck et al. 2006; Kong et al. 2022). Following each iteration of either rough or fine fitting, the weight updating function of TIMESAT *wTSM* (Jonsson and Eklundh 2002), as suggested by *Phenofit*, was utilized to adjust the weighting of each observation within the time series.

**FIGURE 2:**
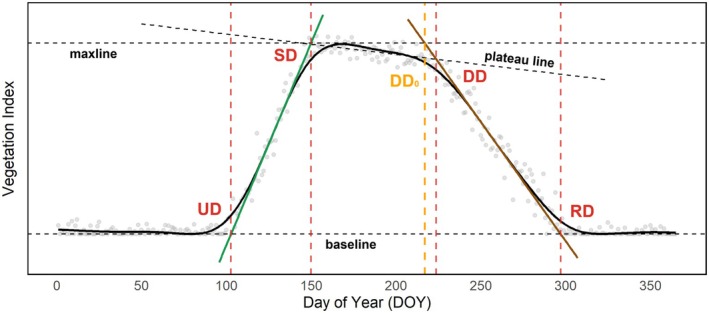
Schematic of LSP extraction in the Gu method. Green and brown lines refer to the recovery line and senescence line, respectively. DD, downturn date; RD, recession date; SD, stabilization date; UD, upturn date.

LSP metrics were extracted from the fine‐fitted reconstructed daily time series using the Gu method, which allows for a finer partitioning of the phenological cycle, ensuring that much more detailed variation information is preserved (Gu et al. 2009). This derivative‐based approach avoids empirical thresholds, thereby reducing human‐induced errors and improving comparability across different vegetation indices and vegetation types. As summarized in Table 2, five phenological metrics were identified: upturn date (UD), stabilization date (SD), initial downturn date (DD_0_), downturn date (DD), and recession date (RD). These metrics are determined by the geometric intersections of tangent lines (recovery and senescence lines) with the baseline, maxline, or plateau line (Figure 2). Notably, to account for the mid‐season greendown effect, the final adjusted DD was determined by the intersection of the senescence line and plateau line (linear fits to VI time series between SD and DD_0_), following the optimization proposed by Kong et al. (2022).

**TABLE 2:** Summary of land surface phenology (LSP) metrics extracted in this study.

Metric	Full name	Biological meaning	Calculation	Variables
UD	Upturn Date	Start of growing season	Recovery line∩baseline	*Baseline and maxline*: the lower and upper boundary of the fitted VI curve. *Recovery line*: Tangent at the max increase rate. *Senescence line*: Tangent at the max decrease rate. *Plateau line*: Linear fit of the peak growth period (between SD and DD_0_).
SD	Stabilization Date	Start of peak growth	Recovery line∩maxline	
DD_0_	Initial Downturn Date	Original end of peak growth	Senescence line∩maxline	
DD	Downturn Date	Adjusted end of peak growth	Senescence line∩plateau line	
RD	Recession Date	End of growing season	Senescence line∩baseline	

### 2.4 Statistical Analysis

The Pearson Correlation (Pearson 1896) was applied to evaluate the relationship of PPI, kNDVI, and EVI with both HCSIF dataset and ground‐based flux tower observed GPP. The correlation coefficient *R* ranges from −1 to 1, with a larger absolute value indicating a stronger correlation. In this study, we calculated within pixel correlations between various VIs and SIF using 8‐day time series at a 500 m spatial resolution. Then, to ensure better match with the tower's footprint, the averaged VI values within a 3 × 3 pixel window were used for correlation analysis against in situ GPP (Rahman et al. 2005; Karkauskaite et al. 2017). GPP was averaged over an 8‐day interval to ensure temporal consistency with the satellite‐derived VI products. Furthermore, since HCSIF data commences on the 60th day of each year, the entire dataset was shifted forward by 3 days during calculations to align with the corresponding time points. We subsequently compared the performance of EVI, kNDVI, and PPI in estimating GPP using simple linear regression models based on tenfold cross‐validation. The performance of each VI was evaluated with coefficients of determination (*R* ^2^), root mean square error (RMSE), and median bias error (Bias) from the linear regression between the observed GPP and estimated GPP.

Trends of LSP derived from three VIs during 2000–2023 were analyzed by two non‐parametric statistical methods. The Theil‐Sen (TS) median trend analysis, notable for its robustness against outliers, does not require the time‐series data to satisfy assumptions of normal distribution and serial autocorrelation (Lavagnini et al. 2011). This method denotes the trend as a rate of change per year by computing the median of slopes between all *n* (*n* − 1)/2 pairwise combinations of the VIs time series data. A positive slope indicates an increasing trend, whereas a negative slope indicates a decreasing trend. The significance of TS median trend is determined by the Mann‐Kendall (M‐K) test (Yang and Fan 2023), calculating the statistic *Z* scores. When |*Z*| > *Z*_1‐α/2_ at a given significance level α, it suggests a significant change in the time series. Specifically, |*Z*| ≥ 1.96 means that the 95% confidence level is passed, and |*Z*| > 2.56 indicates that the 99% confidence level is passed. The TS slope and M‐K test were implemented in Python.

## 3 Results

### 3.1 Relationships of PPI, kNDVI, and EVI With SIF

**FIGURE 3:**
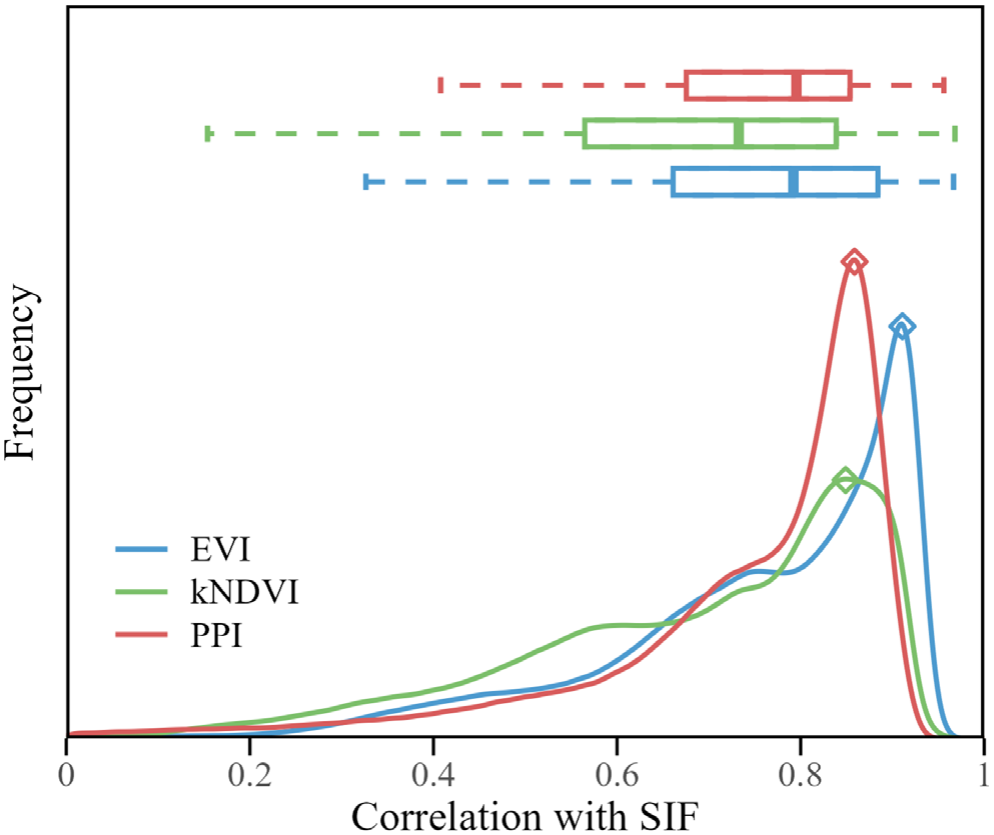
Within pixel correlations between the three VIs and SIF.

All three vegetation indices showed strong positive correlations with SIF, with frequency peaks occurring at correlation coefficients above 0.8 (Figure 3). However, distinct differences existed among them. While EVI had a frequency peak at the highest correlation (*R* > 0.9), PPI exhibited the strongest spatial consistency across the YRB, as evidenced by its highest median correlation coefficient and the narrowest interquartile range. In contrast, the relationship between kNDVI and SIF was weaker and more heterogeneous, characterized by the widest distribution and lowest median in their correlation coefficients.

**FIGURE 4:**
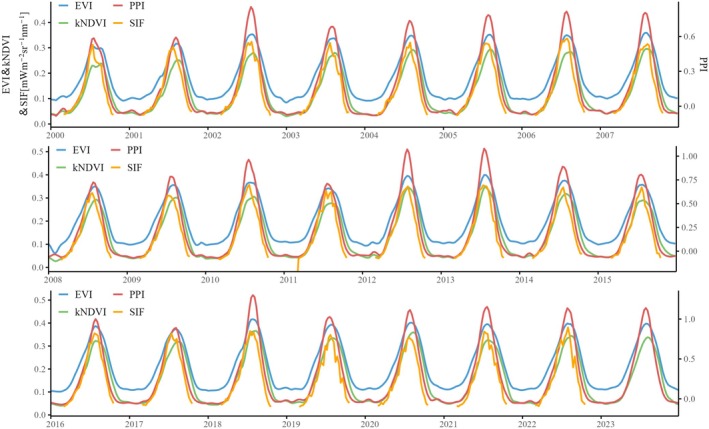
Seasonality of PPI, kNDVI, and EVI in relation to SIF during 2000–2023 (average values calculated for vegetated pixels). Time series is divided into three periods to improve readability.

The seasonality of three VIs and SIF was derived from the average of vegetated pixels in the YRB (Figure 4). PPI exhibited the largest seasonal amplitude, with greater interannual variability in its peak magnitude compared to EVI, kNDVI, and SIF. The average SIF curve was characterized by a less smooth seasonal time series than the other VIs, marked by short‐term fluctuations that were particularly prominent in early 2011. This is likely attributable to the nature of the downscaled product, which is more susceptible to noise and data gaps (Sun et al. 2024). PPI and kNDVI showed good agreement with SIF in terms of the onset of the spring increase. However, PPI had a closer temporal alignment with SIF and peaked earlier, whereas EVI and kNDVI tended to peak later. During the autumn decline phase, SIF consistently declined first, followed by PPI kNDVI and EVI exhibited a similar delayed onset and completion of the seasonal decline.

### 3.2 Relationships of PPI, kNDVI, and EVI With GPP

**FIGURE 5:**
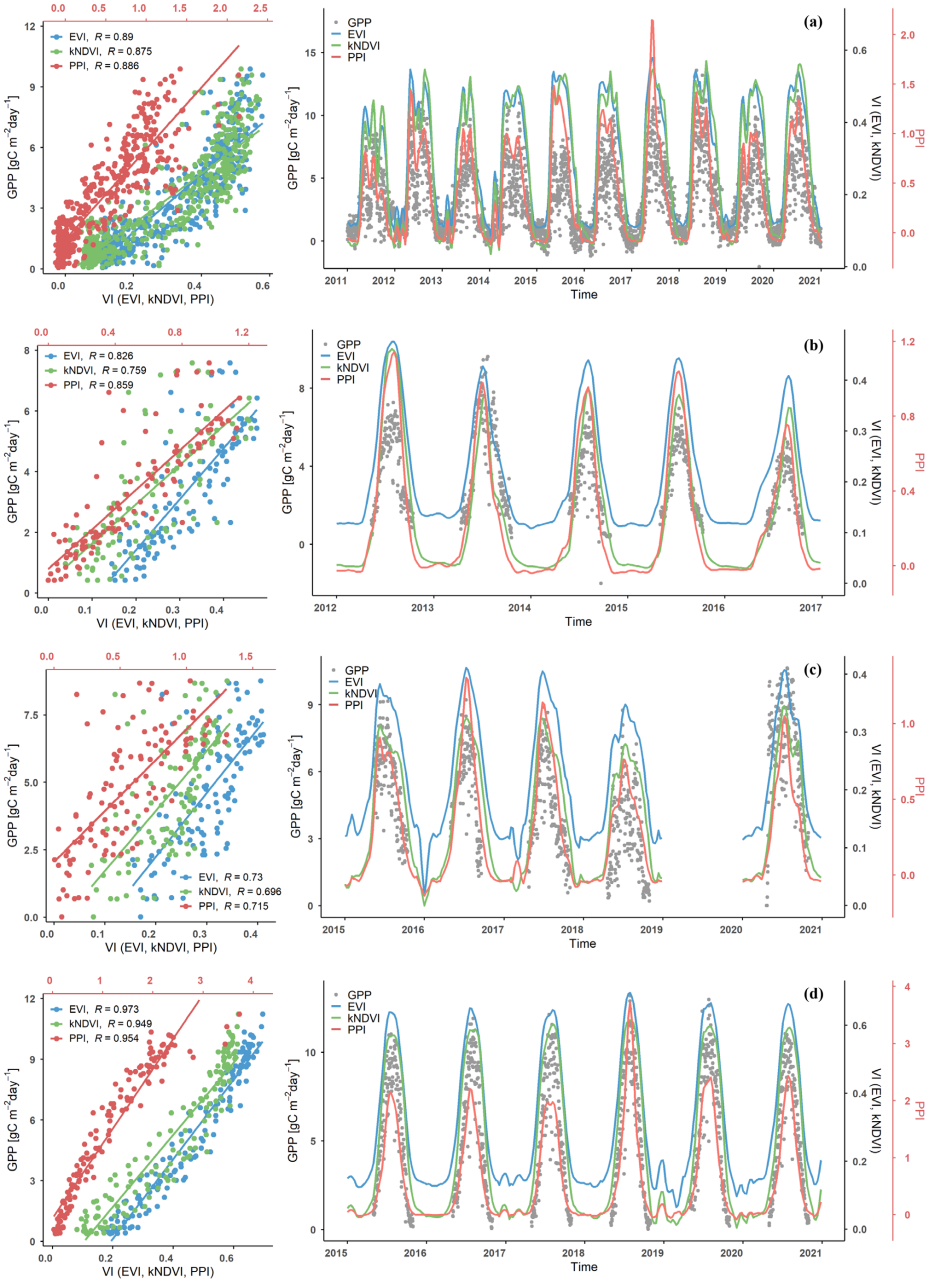
Vegetation indices against GPP observed from four flux tower sites: (a) Xiaolangdi, (b) Sanjiangyuan, (c) Zoige, and (d) Haibei.

To assess which vegetation index more accurately captures the seasonal variations in GPP, we plotted observed GPP from four ground‐based carbon flux towers against the corresponding mean values of each VI within the 3 × 3 pixels (Figure 5). The results indicated that all VIs exhibited the strongest correlation with GPP at the Haibei site, an alpine meadow, with correlation coefficients exceeding 0.9. In contrast, correlations at the Zoige site, also an alpine meadow, were substantially weaker, with coefficients below 0.75 for all VIs. At individual sites, PPI and EVI generally showed slightly stronger correlations with GPP than kNDVI. Notably, PPI was better correlated with GPP (*R* = 0.859) as compared to EVI (*R* = 0.826) and kNDVI (*R* = 0.759) at the Sanjiangyuan flux tower. Regarding the consistency between the temporal dynamics of VIs and the daily distribution patterns of GPP, PPI showed the highest overall agreement with GPP. The characteristic earlier onset of autumn decline observed in the mean PPI time series, relative to EVI and kNDVI, was evident at the pixels level corresponding to flux tower sites and aligned better with the seasonal decline of GPP.

**FIGURE 6:**
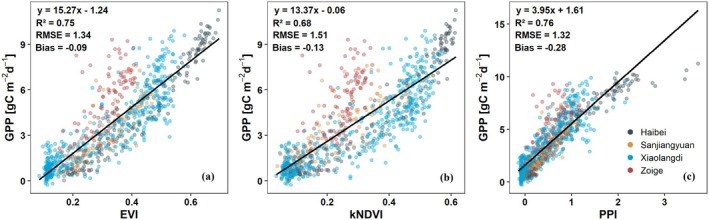
Comprehensive performance of the EVI (a), kNDVI (b), and PPI (c) based on in‐situ GPP data from four flux towers.

Using data from all the flux towers, a linear regression was performed between the 8‐day VIs and GPP (Figure 6). Among all the VIs, the PPI presented the best overall fit, with the highest *R* ^2^ value of 0.76 and the lowest RMSE of 1.31 g C m^−2^ day^−1^. The EVI performed similarly to the PPI, with an *R* ^2^ value of 0.75 and an RMSE of 1.34 g C m^−2^ day^−1^. The kNDVI exhibited the weakest performance, with the lowest *R* ^2^ value of 0.68 and the highest RMSE of 1.51 g C m^−2^ days^−1^. Regarding the overall systematic error, the median Bias for EVI, kNDVI, and PPI were −0.09, −0.13, and −0.28, respectively, indicating a general overestimation across the study sites. Minor site‐dependent biases can be observed, with some stations (e.g., Zoige) tending to lie above the regression line, indicating slight underestimation.

**FIGURE 7:**
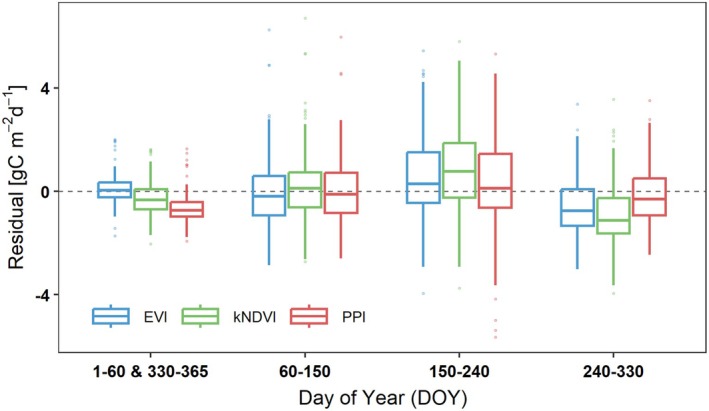
Seasonal distribution of GPP estimation residuals across EVI, kNDVI, and PPI.

Regarding temporal stability (Figure 7), the models showed distinct seasonal behaviors. During the dormant period (1–60 and 330–365 DOY), residuals of all three models were relatively concentrated with small interquartile ranges, indicating stable model performance. However, the median residuals for kNDVI and PPI were predominantly negative, suggesting a tendency to overestimate GPP during this period. On the contrary, all models showed substantial spread during the peak growing season (150–240 DOY), especially PPI, which exhibited the widest dispersion. Median residuals were slightly positive for EVI and kNDVI, indicating mild underestimation. Notably, PPI showed median residuals closest to zero during 60–150, 150–240, and 240–330 DOY, indicating relatively lower bias compared to EVI and kNDVI. This advantage was particularly evident in the late growing season (240–330 DOY), where PPI outperformed the other VIs with a more balanced residual distribution.

### 3.3 Spatial Patterns of Phenological Metrics Based on PPI, kNDVI, and EVI

**FIGURE 8:**
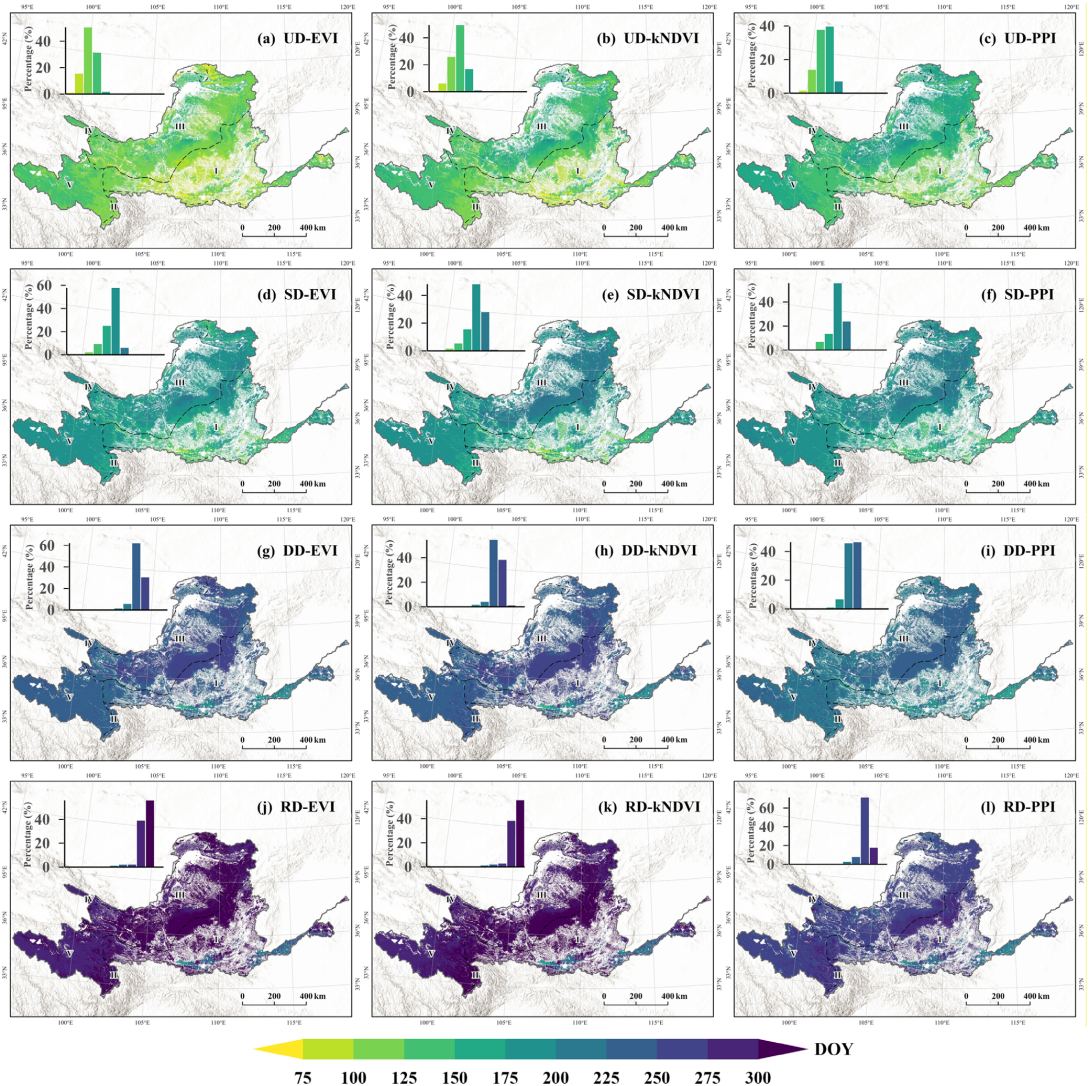
Spatial distribution of mean phenology derived from EVI (a, d, g, j), kNDVI (b, e, h, k), and PPI (c, f, i, l) during 2000–2023. DD, downturn date; RD, recession date; SD, stabilization date; UD, upturn date.

Four phenological metrics, namely UD, SD, DD, and RD, were extracted to characterize vegetation phenology in the YRB from 2000 to 2023 (Figure 8). With respect to the spatial patterns of relative phenological timing, these phenological metrics derived from different VIs showed overall spatial consistency. All three VIs indicated an earlier onset of UD and SD in the southern YRB, and several areas in the far south also underwent a premature senescence phase. Conversely, both the start and end of the growing season were delayed in the northern and central parts of the YRB, especially in vegetation sub‐region III and the transitional areas between sub‐regions I and III. However, discrepancies emerged when comparing the actual day‐of‐year (DOY) values across the three VIs. For instance, UD derived from EVI was mainly concentrated between 100 and 125 DOY, which is significantly earlier than that derived from PPI (150–175 DOY) and kNDVI (125–150 DOY) (Figure 8a–c). Similarly, SD derived from EVI in sub‐region III primarily fell within 175–200 DOY, again slightly preceding the values from kNDVI and PPI (Figure 8d–f). In contrast, DD and RD, two metrics characterizing the decline of the growing season, demonstrated high similarity in spatial distribution when derived from kNDVI and EVI, but occurred approximately 25–50 days later than the corresponding metrics derived from PPI.

### 3.4 Spatiotemporal Trends of Phenological Metrics Based on PPI, kNDVI, and EVI

**FIGURE 9:**
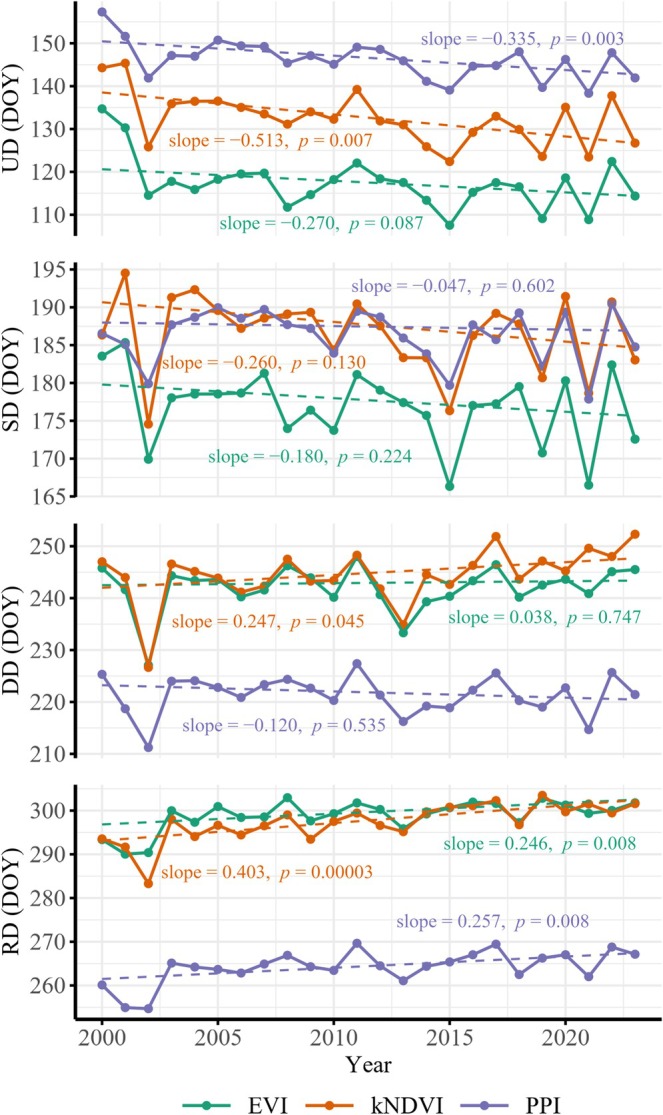
Inter‐annual variations in the mean values of phenology derived from PPI, kNDVI, and EVI during 2000–2023. DD, downturn date; RD, recession date; SD, stabilization date; UD, upturn date.

As illustrated in Figure 9, the basin‐scale averaged UD had a significant advancing trend when extracted from PPI and kNDVI, with rates of −0.335 days year^−1^ (*p* < 0.01) and −0.513 days year^−1^ (*p* < 0.01), respectively. The mean RD across the YRB exhibited an opposite trend, with delays of 0.403, 0.257, and 0.246 days year^−1^ (*p* < 0.01) for kNDVI, PPI, and EVI, respectively. All three VIs indicated an advancing trend in SD, though none of these trends reached statistical significance. Regarding the DD, both kNDVI and EVI revealed delaying trends; however, only the trend based on kNDVI was significant at 0.247 days year^−1^ (*p* < 0.05), while PPI showed a non‐significant advancing trend (−0.120 days year^−1^). When comparing the same phenological metrics across different VIs, kNDVI consistently demonstrated the strongest basin‐wide trends.

According to the results at vegetation sub‐regional scale in the YRB (Table 3), the UD of all three VIs significantly advanced in both vegetation sub‐region I and V. Among them, kNDVI indicated the strongest trend in sub‐region I (−1.046 days year^−1^, *p* < 0.01), while EVI showed a similar trend in sub‐region V (−0.343 days year^−1^). Meanwhile, the SD of all VIs also presented a significant advancing trend in sub‐region I, with kNDVI again indicating the most pronounced shift (−0.759 days year^−1^). It is noteworthy that only the RD of kNDVI delayed significantly across all five vegetation sub‐regions, whereas PPI and EVI showed significant delays exclusively in sub‐region I and III, respectively. Moreover, opposite trends in phenological metrics were observed for PPI compared to kNDVI and EVI in several sub‐regions, such as UD and SD in sub‐region IV, DD in sub‐region I, and RD in sub‐region II, though none of these trends was significant.

**TABLE 3:** Trends of four phenological metrics in different vegetation sub‐regions of the YRB during 2000–2023.

Vegetation sub‐region	Vegetation index	Slope of phenological metrics			
		UD	SD	DD	RD
I	PPI	−0.655**	−0.355*	−0.278	0.233
	kNDVI	−1.046**	−0.759**	0.262	0.400**
	EVI	−0.631**	−0.522**	0.108	0.373**
II	PPI	−0.166	−0.107	−0.243*	−0.073
	kNDVI	−0.292*	−0.032	0.109	0.230**
	EVI	−0.251*	−0.117	−0.019	0.116
III	PPI	−0.232	0.141	0.150	0.343**
	kNDVI	−0.372	0.048	0.389**	0.520**
	EVI	−0.123	0.015	0.099	0.181
IV	PPI	0.008	0.142	0.009	0.103
	kNDVI	−0.115	−0.102	0.040	0.193**
	EVI	−0.016	−0.079	0.016	0.128
V	PPI	−0.217**	−0.197	−0.167	0.067
	kNDVI	−0.267**	−0.234	−0.008	0.212*
	EVI	−0.343*	−0.227*	−0.036	0.095

* *p* < 0.05.

** *p* < 0.01.

**FIGURE 10:**
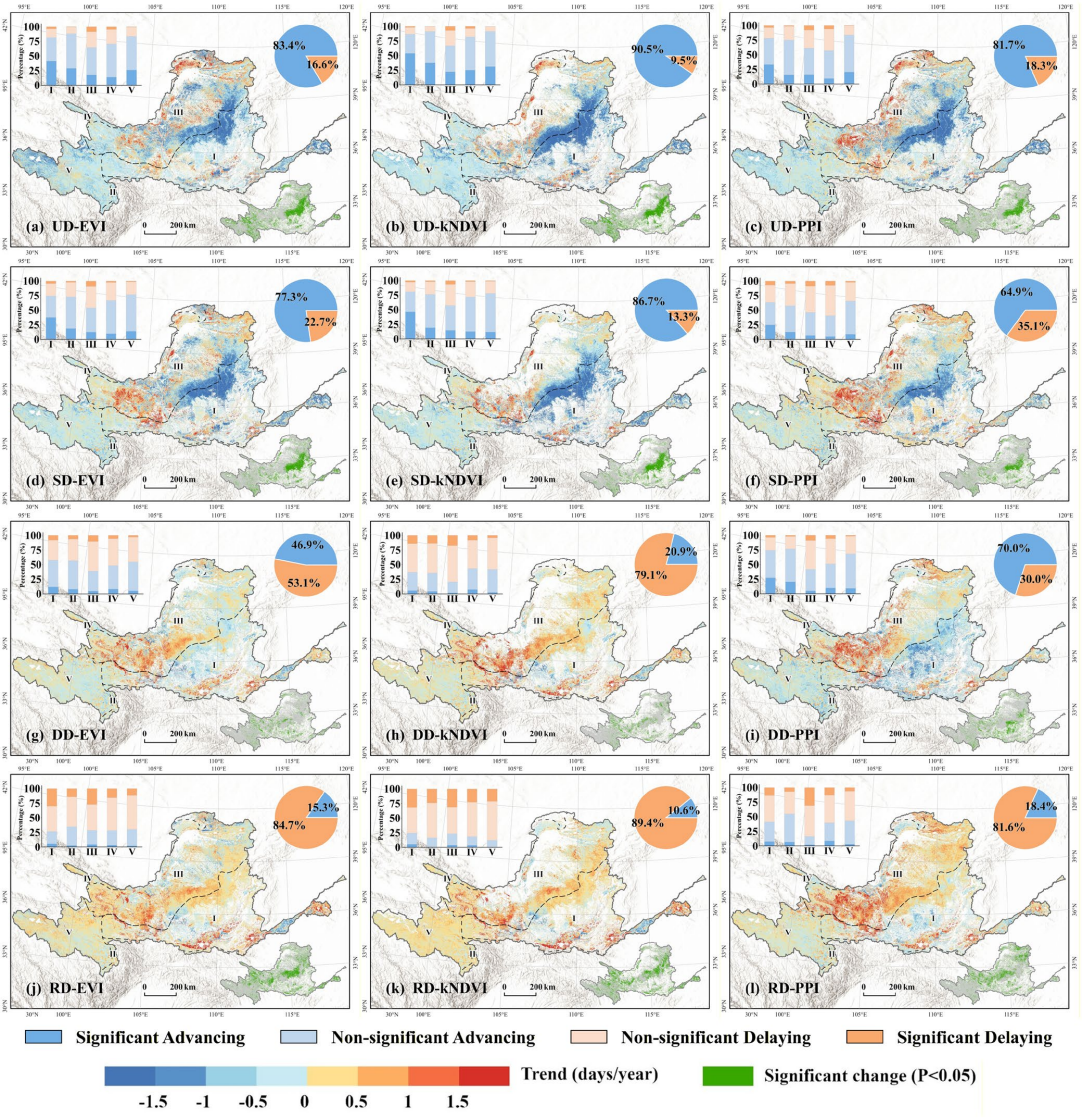
Spatial variations trends in phenology derived from EVI (a, d, g, j), kNDVI (b, e, h, k), and PPI (c, f, i, l) in the YRB and different vegetation sub‐regions during 2000–2023. DD, downturn date; RD, recession date; SD, stabilization date; UD, upturn date.

Spatial heterogeneity was evident in the changes of phenological metrics derived from different VIs across the YRB and its five vegetation sub‐regions (Figure 10). For all VIs, more than 81.7% of the significant pixels for the UD exhibited advancing trends, while over 81.6% of the significant pixels for the RD showed delaying trends. Specifically, the UD displayed strong advancing trends (> 1.5 days year^−1^) in the transitional zones between vegetation sub‐regions I and III, as well as in most parts of sub‐region V in the western basin. In contrast, a limited area in the northwest of sub‐region III presented a marked delaying trend in UD. The spatial pattern of SD trends was somewhat consistent with that of UD, although the percentage of pixels with delaying trends in SD increased for all three VIs. Regarding the RD, all three VIs highlighted significant delaying trends in the west‐central region of the basin (primarily in sub‐region III) and in scattered areas of the southern YRB. However, the DD of PPI presented a distinctly different pattern, particularly in sub‐region I, where a large proportion of pixels indicated significant advancing trends, clearly deviating from the patterns observed for kNDVI and EVI.

## 4 Discussion

### 4.1 Evaluation of Different VIs in Capturing Seasonal GPP Dynamics

As a by‐product of photosynthesis, SIF retrieved from satellite datasets (Jeong et al. 2017; Guanter et al. 2021), imaging spectrometer measurements (Rascher et al. 2015; Bendig et al. 2025), or field experiments (Wu et al. 2024), offers an advantage over greenness‐based VIs in better characterizing actual plant photosynthetic activity (Zhang et al. 2022). Numerous recent studies have confirmed that the relationship between SIF and GPP is strong across spatial and seasonal scales, and robust across various biomes (Zhang et al. 2018, 2022; Li et al. 2018). Consequently, satellite‐based SIF can serve as a benchmark to evaluate the performance of different VIs in capturing GPP variations. In this study, 500 m spatial resolution SIF and in situ tower GPP datasets were employed.

Across the basin, EVI and PPI outperformed kNDVI in terms of correlation with SIF and overall GPP estimation accuracy (Figures 3, 4, 5, 6). Specifically, PPI provided the best overall fit (*R* ^2^ = 0.76, RMSE = 1.31 g C m^−2^ day^−1^), which is consistent with findings by Marsh et al. (2025). They highlight that PPI is superior (average *R* ^2^ = 0.64) to other VIs in northern ecosystems. Comparative analyses also show that PPI outperforms many VIs in capturing seasonal GPP dynamics across ecosystems (Karkauskaite et al. 2017; Tian et al. 2021). The superior performance of PPI and EVI can be attributed to their design. EVI incorporates canopy structure and chlorophyll information (Huete et al. 2002) and has been widely adopted as a proxy for the fraction of photosynthetically active radiation absorbed by vegetation canopies (fPAR) (Liu, Wu, Peng, et al. 2017), which help improve GPP estimation in light use efficiency (LUE) models (Liang et al. 2023). PPI, grounded in radiative transfer theory, can capture within‐canopy light absorption and maintain a linear relationship with LAI (Jin and Eklundh 2014). In contrast, although kNDVI mitigates saturation and mixed‐pixel effects, it primarily reflects structural greenness rather than photosynthetic efficiency (Camps‐Valls et al. 2021), leading to weaker correlations with GPP, particularly in woody‐dominated systems (Wang et al. 2022). Tian et al. (2026) further clarified this performance gap by grouping VIs into two categories: NDVI and kNDVI, which track green chromatic coordinate (GCC) and reflect structural greenness, and EVI2, NIRv, and PPI, which better correspond to LAI and flux tower GPP.

Besides, the divergent performance of VIs under extreme conditions, such as the extensive snow cover (Li et al. 2023) at the Sangjiangyuan and Zoige sites (Figure 5b,c), highlights their varying robustness to background noise. PPI exhibited a stronger correlation with GPP in these regions, likely due to its documented insensitivity to snow contamination (Jin and Eklundh 2014), as also reported in subsequent studies (Karkauskaite et al. 2017; Marsh et al. 2025). EVI has also shown good capability in mitigating atmospheric and soil background influences on vegetation monitoring through incorporating the blue band and background adjustment factor (Huete et al. 2002). However, limited knowledge is available regarding the resistance of kNDVI to background noise. This is consistent with Zeng et al. (2025), who reported better kNDVI performance in deciduous broadleaf forest (DBF) and evergreen needle‐leaf forest (ENF), which are generally less affected by background interference.

Furthermore, the temporal consistency analysis revealed that PPI better captured the autumn senescence compared to EVI and kNDVI (Figures 4 and 7). This earlier decline in PPI aligns more closely with SIF and GPP, reflecting the physiological reality that photosynthetic activity often wanes before substantial structural changes occur in the canopy (Li et al. 2018). Camps‐Valls et al. (2021) further noted that while photosynthesis is influenced by the quantity of photosynthetically active biomass within a pixel, it is also strongly regulated by incoming solar radiation and environmental constraints, variables that the NIR‐red spectral signal alone cannot capture. PPI's closer tracking of the autumn downturn may therefore reflect its built‐in correction for solar‐zenith angle and its robustness to snow contamination (Jin et al. 2017). Nevertheless, PPI exhibited increased sensitivity to noise during peak growing seasons (Figure 5a), a limitation previously reported by Jin and Eklundh (2014). This noise sensitivity might partially account for its occasional lower correlation with GPP compared to EVI.

Taken together, the three vegetation indices exhibit a certain degree of complementarity in capturing the seasonal dynamics of GPP. kNDVI performs well during the early growing season, whereas EVI shows the most stable overall performance, particularly during peak biomass periods, thereby helping to compensate for the higher peak‐season variability observed in PPI. In contrast, PPI performs better during transitional periods, especially in capturing the timing of autumn senescence. These complementary characteristics suggest that no single index performs optimally across all stages of vegetation development. Therefore, integrating multiple indices may provide a more comprehensive representation of vegetation photosynthetic dynamics, particularly in heterogeneous ecosystems such as the Yellow River Basin. Such a multi‐indices perspective also facilitates disentangling the relative contributions of structural and physiological controls on vegetation dynamics, providing additional insights into ecosystem functioning and its response to environmental variability.

### 4.2 Consistency and Disparity in VI‐Based Phenological Metrics Across the YRB

Among the PPI, kNDVI, and EVI, there was a broad agreement in capturing the spatial and temporal variations of vegetation phenology across the YRB. Spatially, the UD and SD derived from PPI and kNDVI progressively advanced from the southern to the western parts of the basin, and then to the central‐northern regions (Figure 8), where the land cover was primarily characterized by grasslands (Figure 1b). In contrast, the EVI‐based estimate was more consistent with previous results derived from NDVI, indicating that the central and western regions green up almost simultaneously, with the central region sometimes slightly earlier (Wang et al. 2019; Yuan, Wang, et al. 2020). These findings suggest that in the grassland‐dominated ecosystems across the YRB, PPI and kNDVI are more coherent in capturing the onset of vegetation growth, thereby depicting a similar pattern of spatial heterogeneity. For the end of the growing season, all three VIs exhibited a generally consistent pattern. Only a few scattered regions in the southeast exhibited an earlier RD than the west, whereas the high‐elevation areas in the west senesced earlier than the central basin, which is in accordance with previous studies on SIF and NDVI based estimations of autumn phenology in the YRB (Yuan, Zhao, et al. 2020; Wang et al. 2023).

Temporally, the basin‐mean UD advanced significantly for PPI and kNDVI, while RD was significantly delayed across all three VIs (Figure 9). This result aligned with existing research using NDVI at broader temporal scales (1982–2015) (Yuan, Wang, et al. 2020; Yuan, Zhao, et al. 2020). Furthermore, the spatial distribution of phenological trends revealed that UD advanced strongly along the boundary between vegetation sub‐region I and III, but only weakly in the high‐altitude areas of the western basin (Figure 10). Conversely, RD exhibited significant delays in the southern part of sub‐region III and scattered areas of sub‐region I. Although these spatial trends are consistent with earlier findings, the magnitudes of change are noticeably larger. This is likely attributable to the finer spatiotemporal resolution of current data and the refined classification of phenological metrics. In other words, the UD advance and RD delay observed in this study appear more pronounced than the previously documented trends in the start (SOS) and end of the growing season (EOS) across the YRB.

Disparities in phenological results derived from three VIs were also evident across the YRB, reflecting their sensitivity to different aspects of vegetation dynamics. At the basin scale, the UD and SD extracted from EVI were significantly earlier by 25–75 days than those from PPI and kNDVI, while the DD and RD from PPI were consistently 25–50 days earlier than the other two indices (Figure 8). This aligns closely with the significantly earlier decline in the PPI time series during autumn senescence. In addition, the spatially consistent pattern of SD derived from PPI and kNDVI suggests that both indices are more effective than EVI at mitigating saturation effects. Han et al. (2025) found that kNDVI excels at detecting maize tasseling stage, while Bellini et al. (2023) reported its reliability for estimating SOS and peak of the growing season (POS), yet noted its weaker performance in identifying EOS. These findings indirectly support our suggestion that PPI is superior to kNDVI or EVI in capturing autumn phenology. Moreover, when comparing the same phenological metric, kNDVI showed the strongest basin‐wide trends and the largest fraction of pixels with the prevailing trend direction (advancement or delay). This implies that phenological shifts detected by kNDVI tended to be more pronounced or spatially extensive than those captured by PPI and EVI. Whether kNDVI behaves consistently in other regions, and the underlying mechanisms driving this behavior, warrant further systematic investigation.

Overall, the observed consistency and divergence among VI‐based phenological metrics highlight that different indices capture complementary dimensions of vegetation phenology. Structural indices such as kNDVI emphasize canopy development and greenness dynamics, whereas indices related to photosynthetic processes such as PPI and EVI better reflect functional activity and carbon uptake. Ecologically, this multi‐index and multi‐metric analysis helps disentangle structural and physiological controls on vegetation dynamics. In particular, multiple phenological indicators allow a more detailed characterization of key transitional stages, improving understanding of ecosystem responses to environmental variability. The spatial heterogeneity across the YRB further suggests that these controls vary between vegetation types and along environmental gradients, with implications for ecosystem adaptation and climate change response.

### 4.3 Limitations

Several limitations of this study should be noted. First, the limited data availability and insufficient number of ground‐based observation stations have restricted the spatial and temporal coverage of in situ GPP measurements across the YRB, which may introduce uncertainties into the results. Also, a scale mismatch exists between MODIS 500 m pixels and flux tower observations can not be ingnored, which may introduce uncertainty due to sub‐pixel heterogeneity in land cover and vegetation structure. Although digital cameras such as the PhenoCam network now provide accessible imagery for validating satellite‐based phenology at regional scales (Javadian et al. 2025), a globally long‐term phenology observation system with unified standards for definitions and measurements remains urgently needed. Second, we only adopted a single method to extract phenological metrics and did not evaluate the performance of alternative extraction approaches across different VIs. Further research should therefore assess the accuracy and applicability of various phenology extraction algorithms for different VIs, and develop integrated models that synergistically leverage multiple VIs to enhance estimation accuracy. Moreover, incorporation of machine learning and deep learning techniques promises to better capture vegetation‐phenology dynamics and improve the accuracy of phenological forecasts.

## 5 Conclusions

This study comparatively assessed the performance of two novel VIs (i.e., PPI, kNDVI) and a traditional one (i.e., EVI) for LSP estimation across the YRB and its five vegetation sub‐regions. While all VIs generally tracked photosynthetic dynamics, EVI demonstrated a stronger overall correlation with SIF, whereas PPI and kNDVI were more consistent with SIF at the onset of spring green‐up. PPI achieved the best overall GPP fit (*R* ^2^ = 0.76, RMSE = 1.31 g C m^−2^ day^−1^), particularly in seasonally snow‐covered alpine regions like Sanjiangyuan (*R* = 0.859). PPI also showed more balanced and stable errors during late‐season GPP estimation. Significant differences were observed in phenological metrics across the basin and vegetation sub‐regions, especially in autumn phenology. DD and RD derived from kNDVI and EVI were spatially consistent but occurred 25–50 days later than those from PPI. All VIs showed advances in UD and SD and delays in RD, but diverged in DD, with PPI indicating earlier DD across 70% of the basin compared to delayed estimates from kNDVI (79.1%) and EVI (53.1%). In grasslands, PPI and kNDVI were more consistent in spring phenology, whereas stronger divergence appeared in forest ecosystems during autumn. Our results highlight that PPI is more sensitive to autumn phenology than kNDVI and EVI, offering a promising option for detecting the transition dates of autumn senescence.

## Author Contributions

**Qinyue Yu:** conceptualization (equal), data curation (equal), methodology (equal), visualization (equal), writing – original draft (equal). **Yan Bai:** formal analysis (equal), resources (equal), supervision (equal), writing – review and editing (equal). **Juanle Wang:** supervision (equal), writing – review and editing (equal).

## Funding

This work was supported by the National Key Research and Development Program of China (2024YFF0729005), National Earth System Science Data Center (2005DKA32300), and Earth System Data Center of the Chinese Academy of Sciences (CAS‐WX2022SDC‐XK18).

## Conflicts of Interest

The authors declare no conflicts of interest.

## Data Availability

The PPI, kNDVI, and EVI data were calculated from the MODIS NBAR daily product (MCD43A4, v061, 500 m) on Google Earth Engine (https://developers.google.com/earth‐engine), the HCSIF data were obtained from https://doi.org/10.1038/s41597‐024‐04101‐6, in situ GPP data were obtained from ChinaFLUX (https://www.chinaflux.org/); MODIS land cover data (MCD12Q1, v061, 500 m) were obtained from NASA EOSDIS (https://www.earthdata.nasa.gov/); the DEM data were obtained from the ASTER GEDM (https://lpdaac.usgs.gov/), the vegetation sub‐region data were obtained from the NESSDC (http://www.geodata.cn/). R code for phenology retrieval and analysis are publicly available at Zenodo (https://doi.org/10.5281/zenodo.18459429).
